# Targeting acute myeloid leukemia stem cell signaling by natural products

**DOI:** 10.1186/s12943-016-0571-x

**Published:** 2017-01-30

**Authors:** Kodappully Sivaraman Siveen, Shahab Uddin, Ramzi M. Mohammad

**Affiliations:** Translational Research Institute, Academic Health System, Hamad Medical Corporation, PO Box 3050, Doha, Qatar

**Keywords:** Acute myeloid leukemia, Leukemia stem cells, Self-renewal, Natural products

## Abstract

Acute myeloid leukemia (AML) is the most commonly diagnosed leukemia in adults (25%) and comprises 15–20% in children. It is a genetically heterogeneous aggressive disease characterized by the accumulation of somatically acquired genetic changes, altering self-renewal, proliferation, and differentiation of hematopoietic progenitor cells, resulting in uncontrolled clonal proliferation of malignant progenitor myeloid cells in the bone marrow, peripheral blood, and occasionally in other body tissues. Treatment with modern chemotherapy regimen (cytarabine and daunorubicin) usually achieves high remission rates, still majority of patients are found to relapse, resulting in only 40–45% overall 5 year survival in young patients and less than 10% in the elderly AML patients. The leukemia stem cells (LSCs) are characterized by their unlimited self-renewal, repopulating potential and long residence in a quiescent state of G_0_/G_1_ phase. LSCs are considered to have a pivotal role in the relapse and refractory of AML. Therefore, new therapeutic strategies to target LSCs with limited toxicity towards the normal hematopoietic population is critical for the ultimate curing of AML. Ongoing research works with natural products like parthenolide (a natural plant extract derived compound) and its derivatives, that have the ability to target multiple pathways that regulate the self-renewal, growth and survival of LSCs point to ways for a possible complete remission in AML. In this review article, we will update and discuss various natural products that can target LSCs in AML.

## Background

AML is a malignant, heterogeneous, clonal disease arising in hematopoietic stem or progenitor cells resulting from genetic and epigenetic alterations that perturb key processes like self-renewal, proliferation and differentiation. It is characterized by malignant clonal proliferation and/or differentiation of immature myeloid progenitor cells [1, 2]. In AML, the myeloid stem cells differentiate to malignant myeloblasts that cannot function as normal blood cells. Consequently bone marrow and peripheral blood gets accumulated with leukemic blasts resulting in significantly reduced production of healthy and functional white blood cells, red blood cells, platelets, and mature granulocytes.

AML is one of the most common hematological malignancy. The other common hematological malignancies includes Lymphoma (Hodgkin lymphoma and Non-Hodgkin lymphoma), Myeloma, Leukemia (acute lymphocytic leukemia, chronic lymphocytic leukemia and chronic myeloid leukemia), Myeloproliferative neoplasms (essential thrombocythemia, polycythemia vera and myelofibrosis) and Myelodysplastic syndromes (refractory anemia, refractory anemia with ring sideroblasts, refractory anemia with excess blasts, refractory anemia with excess blasts in transformation and chronic myelomonocytic leukemia). In United States, as per American Cancer Society, an estimated 60,140 new cases of leukemia are expected in 2016 and 24,400 deaths resulting from leukemia. This includes 6,590 new cases of acute lymphocytic leukemia and 1,430 deaths, 18,960 new cases of chronic lymphocytic leukemia and 4,660 deaths, 19,950 new cases of acute myeloid leukemia and 10,430 deaths, and 8,220 new cases of chronic myeloid leukemia and 1,070 deaths [3].

AML is the most common form of acute leukemia in adults and constitutes approximately 80 percent of cases. The incidence of AML is about 1.3 per 100,000 for those under 65 and about 12.2 cases per 100,000 for those over 65 years [4]. There is significant patient-to-patient heterogeneity within the morphologic-genetic characteristics of leukemic blasts. Based on the morphologic heterogeneity, AML is classified into seven French–American–British (FAB) subtypes. A common feature of AML is the arrest of aberrant differentiation leading to accumulation of more than 20% blasts in the bone marrow [5]. Mutations in genes involved in regulation of cell proliferation (Fms-like tyrosine kinase 3, c-KIT, RAS etc.,) and apoptosis (nucleophosmin, p53 etc) are used as a prognostic factor of clinical outcome in AML. These genetic alterations are potential therapeutic targets, but targeting them have failed to show any sufficient activity against the various cell types present in AML [6].

The present chemotherapeutic approach for the management of the disease is based on the concept of targeting leukemic cells specifically to eradicate them while minimally affecting normal cells. The induction therapy with cytarabine in combination with new generation anthracycline antibiotics (daunorubicin/Epirubicin/Idarubicin/Mitoxantrone) is the back bone of AML treatment [7], and has significantly improved the rate of remission in AML over the years, but more than 50% relapse with resistant disease [8], causing the death of most patients, remains a major hindrance in a successful AML chemotherapy [9].

## AML stem cells

Acute myeloid leukemia stem cells/leukemia initiating cells (LSCs) were defined as those cells capable of regenerating human AML cell populations in irradiated non-obese diabetic/severe combined immunodeficient (SCID) mice after transplantation [10, 11]. LSCs possessing this renewal property were found to display a CD34^+^CD38^−^ phenotype, which is similar to normal human hematopoietic progenitors population [12, 13]. The existence of LSCs which are characterized by their self-renewal capability, unlimited repopulating potential and prolonged residence in the G_0_/G_1_ phase of cell cycle in a quiescent state was initially pointed out by Lapidot et al. [11]. Limitless self-renewal is an important property of cancer stem cells that is distinct from tumor cell proliferation. The self-renewing cell division of LSCs can result in one/both daughter cells that have the ability for further self-renewal and/or generate differentiated progenitor blast cell lineages, in a way that is highly analogous to normal hematopoietic stem cells [14]. A crucial aspect of AML is that the tumor population is heterogeneous and that LSCs are biologically distinct from the highly differentiated blast cells [15]. Further studies on the immunophenotype of LSCs have shown that these cells can be defined as expressing CD34, CD117, CD123, CD382, CD902 and HLA-DR2 [16]. Some of these markers are also expressed in hematopoietic stem cells (HSCs), but CD123, CD47, CD44, CLL-1, CD96, CD90, CD32, CD25, and TIM-3 is reported to be leukemia-specific [17].

For over 10 years, the backbone of AML therapy has remained same, with an initial remission induction therapy followed by several months of consolidation therapy. The initial remission induction uses a combination of nucleoside analogs drugs (eg, cytosine arabinoside) and anthracyclines antibiotics (eg, idarubicin, daunorubicin) that interfere with DNA replication to induce apoptosis primarily in replicating cells, while the consolidation therapy consists of cytosine arabinoside in multiple cycles [15, 18]. Since LSCs maintain a largely quiescent cell cycle status, conventional drugs are unlikely to affect the stem cell population, as the LSCs are primarily in G_0_ phase of cell cycle; LSCs population may also possess natural mechanisms of survival (drug efflux pumps, multi-drug resistance (MDR)) as they are developmentally primitive than tumor cells; and because the LSCs are biologically similar to normal cells as they have fewer oncogenic lesions, thus less susceptible to tumor-specific drugs [19].

Chemotherapeutic treatment of AML can result in complete remission in most cases, but relapses frequently occur [20], leading to an overall survival of only 30% to 40% at 4 years after diagnosis [21]. A high relapse rate suggests that current therapies spare the LSCs in AML, and point out the role of this compartment that forms the reservoir for subsequent relapse and resistance. These leukemic stem cells are also being considered to be the initiator of the malignancy, that is often resistant to standard chemotherapy drugs. Even a small number of LSC that survive the initial induction chemotherapy can either lead to drastic reduction in overall survival if they expand rapidly after the end of treatment cycle or lead to relapse if they remain dormant after cessation of therapy [22].

The abundance of LSCs has been associated with clinical relapse or refractory disease [21]. Evidence from recent studies points out that AML is maintained by a population of LSCs, which is insensitive to conventional chemotherapy and have a central role in the relapse of AML [23]. Stem cell frequency at diagnosis offers a new prognostic factor in AML, and a large CD34^+^CD38^-^ population reflects a higher percentage of chemotherapy-resistant cells that will lead to the outgrowth of minimal residual disease, thereby affecting clinical outcome [21]. ATP-binding cassette (ABC) transporters are transmembrane proteins capable of exporting a wide variety of chemotherapeutic drugs from the cytosol and play a major role in conferring multidrug resistance to the host cells. Normal HSC are known to express high levels of surface membrane proteins involved in drug resistance such as MDR1 [24] and BCRP1/ABCG2 (ABC transporters associated with drug resistance) [25] that function to efflux certain molecules. Chemotherapeutic drugs such as anthracyclines are known to be substrates for these efflux pumps and is readily removed from these cells relatively fast. LSCs also express resistant-related proteins like MDR1 and BCRP1 for their multidrug-resistant characters [26].

LSCs share many characteristics with normal hematopoietic stem cells (HSCs), such as a hierarchical developmental pattern, a mostly quiescent state, display heterogeneity within the stem cell compartment and an immunophenotype similar to HSC (CD34^+^, CD38^-^, CD71^-^, and HLA-DR^-^) [16], but at the same time can be phenotypically distinguished from HSCs with of aberrant expression of several distinct (ex. CD123) and sporadically occurring markers among individual patients. Due to the presence of a large set of common features, it has been extremely difficult to elucidate strategies to differentially target the LSC population while sparing HSC. Despite that, recent publications show that LSC population still display some unique molecular properties such as constitutive activation of nuclear factor κB (NF-κB), expression of CD123, and potentially elevated levels of interferon regulatory factor 1 (IRF-1) and death-associated protein (DAP) kinase [27]. These features define LSC population as a critical target in AML therapy and suggests that LSC-selective therapies that spare the hematopoietic stem cell population should improve treatment outcomes in AML.

## Maintenance of LSC population

The pro-inflammatory transcription factor, NF-κB, is known to have antiapoptotic activity and its regulated gene products play a critical role in the proliferation, survival and chemoresistance [28]. Many studies have shown that the NF-κB signaling pathway plays an important role in the drug resistance of tumor cells and many chemotherapeutic drugs and radiotherapy induce NF-κB expression in vitro and in vivo [29]. NF-κB has been found to be constitutively activated in human AML stem cells while normal human CD34^+^ progenitor cells do not express NF-κB [30]. Targeting this aberrant expression may induce apoptotic stimulus and/or sensitize LSC to a variety of other agents and thus can be used to target LSC without significant toxicity to normal hematopoietic stem cells (HSC) [31]. Strikingly, the commonly used AML chemotherapy agents (nucleoside analogues and anthracyclines) does not inhibit NF-κB, instead leads to further upregulation of NF-κB activity [32–34] .

The PI3K/Akt/mTOR pathway is a key signaling cascade in mammalian cells, which regulates mRNA translation of genes that encode for pro-oncogenic proteins, leading to malignant cell survival in various cancers [35]. Constitutive and cytokine-mediated activation of PI3K/Akt/mTOR signaling pathway is a common feature in AML patients, and inhibition of this pathway is a viable therapeutic strategy in the treatment of AML [36]. Xu et al. [37] and Zhao et al. [38] have reported that Akt, a critical substrate of PI3 kinase, is activated in AML blasts, and there is a dose-dependent decrease in survival of most AML patient samples after incubation with the PI3 kinase inhibitor LY294002, while normal hematopoietic progenitors were less affected, suggesting preferential targeting of leukemia cells. The downstream targets of PI3K-Akt include the proapoptotic protein BAD, caspase-9 and NF-κB. Under some circumstances, Ras/PI3K -mediated pathway is already known to activate NF-κB, which suggests a common survival pathway of LSCs triggered by both factors [39]. Birkenkamp et al. [40] have reported that NF-κB was constitutively activated in 73% of AML cases, and the activation status was associated with resistance to spontaneous apoptosis. Treatment of these primary AML cells with the PI3 kinase inhibitor LY294002 and Ras inhibitor L-744832 resulted in downregulation of NF-κB DNA binding activity.

The tumor suppressor gene, PTEN (phosphatase and tensin homologue) is the major negative regulators of the PI3 kinase pathway, which regulates diverse cellular processes, including growth, survival and proliferation of LSCs. PTEN modulates PI3 kinase pathway by dephosphorylating the intermediary PIP3, lipid-signaling molecule [41]. It is also one of the most frequently mutated proteins in human cancers, leading to constitutive activation of PI3 kinase signaling pathway [42]. Deletion of PTEN promotes HSC proliferation leading to HSC depletion through a cell-autonomous mechanism, and generation of transplantable leukaemia-initiating cells. As a result, HSCs is unable to maintain themselves without PTEN, while LSCs proliferation and self-renewal are enhanced by PTEN deletion. Treatment of LSCs with rapamycin was found to deplete leukaemia-initiating cells while restoring normal HSC function, proving that these effects were mostly mediated through mTOR [43].

The JAK-STAT signaling pathway regulates a variety of biological functions, including hematopoiesis, immune-regulation, fertility, lactation, growth and embryogenesis, throughout development [44]. STATs are constitutively activated in several solid tumors and hematological malignancies, including AML [45, 46]. Activation of STATs provides a growth advantage to tumor cells, allowing accumulation, and also confers resistance to conventional therapies that rely on apoptotic machinery to eliminate tumor cells [47]. An increase in STAT3 and STAT5 phosphorylation was reported in AML blasts suggesting vital role of JAK/STAT signaling pathway to support AML stem cell growth and survival [48].

The p53 gene is wild type in more than 90% of AML patient samples [49]. Primary AML cells treated with proteasome inhibitors and the anthracycline idarubicin was found to induce activation of p53 along with increase in the levels of p53 target genes GADD45, p21, and Bax, all of which are strongly implicated in p53-mediated apoptosis [50]. Hence, strategies involving activation of the p53 mediated pathway can be utilized in the majority of AML patients.

Wnt/beta-catenin, Hedgehog and Notch signaling pathways are all involved in the regulation of HSC self-renewal mechanisms and these pathways are dysregulated in leukemic stem cells. There are crosstalks between Wnt/beta-catenin, Hedgehog and Notch signaling, and the PI3K/Akt pathway. Glycogen synthase kinase-3β, involved in canonical Wnt signaling, regulates the molecules involved in Hh signaling, while the pathological response to oncogenic Hh signaling is dependent on Wnt signaling pathway. The PI3K/Akt survival signaling pathway regulates the stem/progenitor cells by promoting Wnt/beta*-*catenin pathway through phosphorylation of GSK-3β, thereby stabilizing β-catenin [51].

The Hedgehog (Hh) signaling pathway is important in the regulation of stem/progenitor cell expansion, cell differentiation, tissue polarity, cell proliferation and tissue repair [52]. Hh regulates hematopoietic stem/progenitor cells via stromal cells. Abnormal Hh pathway activation occurs in several human cancers, including AML, where Hh signaling promotes the maintenance of LSCs and enhance resistance to chemotherapeutic agents [53]. Aberrant activation of Hh signaling is involved in a variety of cancers including AML [54], and is required for the maintenance of the LSC population [55]. Hh signaling pathway is active in primary CD34^+^ LSCs and cytokine-responsive CD34^+^ cell lines (Kasumi-1, Kasumi-3 and TF-1) and contributes to the survival and drug resistance of CD34^+^ leukemia stem cells [56]. AML derived stromal cells had markedly lower expression of hedgehog-interacting protein than healthy donor-derived stromal cells and was found to support the proliferation of SMO^+^ leukemic cells [57].

The Wnt/beta-catenin (Wingless) pathway modulates self-renewal, proliferation, differentiation and apoptosis. Wnt proteins are a diverse family of lipid-modified glycoproteins that bind to Frizzled receptors and lipoprotein receptor-related protein -5/6 coreceptors. Aberrant activation of Wnt pathway by genetic and epigenetic changes is prominent in the initiation and progression of AML. Overexpression of beta-catenin is commonly found in AML samples and is an independent adverse prognostic factor [58]. The Wnt/β-catenin pathway is normally active in HSCs, but β-catenin is not essential for self-renewal of HSCs. Constitutively active β-catenin cooperates with HoxA9/M to induce AML from non-self renewing granulocyte/macrophage restricted progenitors cells, and pharmacological inhibition of β-catenin was found to impairs LSCs formation [59]. Hence targeting the Wnt/β-catenin pathway represents a viable therapeutic option in AML.

Notch signaling plays a crucial role in development, stem cell self-renewal and hematopoiesis. The target genes of Notch involved in cell cycle regulation (cyclin D1, cyclin A , p21, p27), cell proliferation and survival (c-myc, NF-κB2 , Akt, mTOR), embryonic development (Hes1, Hes6), angiopoiesis (VEGF, VEGFR-2), invasion and metastasis (MMP-9, MMP-2) [60]. Deregulation of Notch signaling has been reported in multiple human hematological malignancies, including AML [61]. Human AML samples have extremely low levels of activated Notch receptor and expression of downstream targets, even with a robust expression of Notch receptors, suggesting that Notch is not constitutively activated [62]. In AML initiating cells, activation of Notch was found to inhibit AML growth and survival in vitro and in vivo, which involved caspase-mediated apoptosis driven by Bcl-2 and p53 in response to induction of Notch signaling [63, 64].

Similarly, the polycomb group (PcG) repressor protein Bmi-1 has been shown to mediate self-renewal of both HSC [41] and leukemic stem cells. Bmi-1 is overexpressed in AML cells and is associated with an unfavorable prognosis [65]. Repression of Bmi-1 in normal and leukemic CD34^+^ AML cells by a lentiviral RNA interference approach impairs self-renewal and induces apoptosis [66]. Data from all these studies supports the concept that basic mechanisms of self-renewal are shared between HSCs and malignant LSCs.

MicroRNAs are naturally occurring 18 to 25 nucleotide RNAs that can hybridize to specific target messenger RNA and repress their translation into proteins by controlling endogenous mRNAs at the post-transcriptional level. Several recently publications demonstrated that microRNA levels are altered in AML and correlates with clinical outcome [67]. miR-126 was reported to regulate distinct self-renewal outcomes in normal hematopoietic stem cells and LSC population from AML patients. AML stem cell fraction generally expressed high levels of miR-126 and is associated with poor survival and higher chance of relapse. Overexpression of miR-126 was found to maintain LSCs in a more primitive state by increasing quiescent cell population and increase chemoresistance, while knockdown results in reduced cell growth by inducing apoptosis in vitro and in vivo [68, 69]. PI3K/Akt/mTOR pathway was found to be targeted by miR-126 [70]. miR-9 was reported to promote proliferation of AML stem cells via negative regulation of Hes1 expression by interacting with 3′-untranslated region of Hes1 mRNA. Knockdown of miR-9 can inhibit the AML stem cell proliferation in vitro and increase the survival in a xenotransplant mouse model [71].

**Fig. 1:**
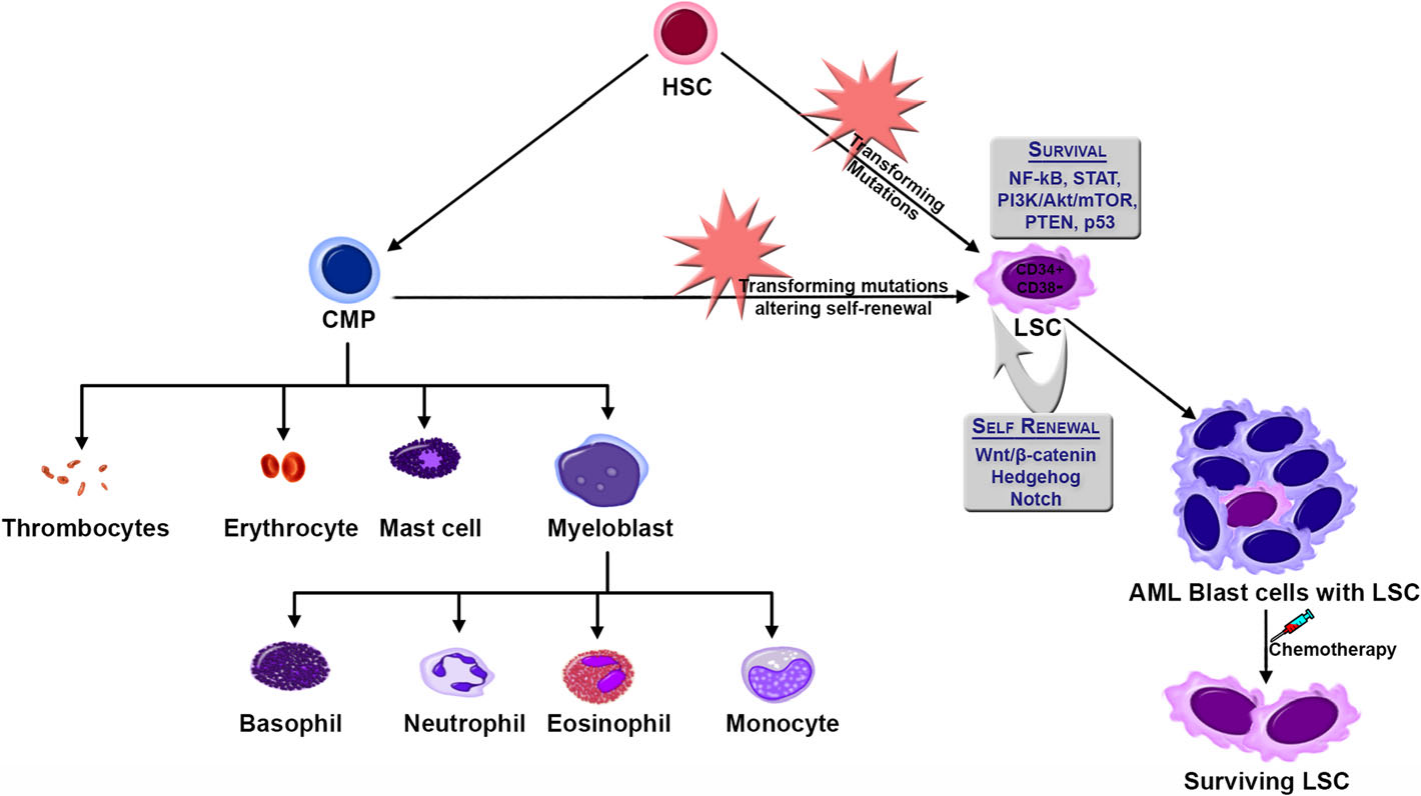
Leukemia stem cells in AML. Transforming mutations in hematopoietic stem cells (HSC) and/or common myeloid progenitor (CMP) leads to the formation of leukemia stem cells (LSCs), which have mutations in survival signaling pathways and altered self renewal capacity. Chemotherapy can significantly abolish the AML blast cells population while the LSCs survive, and instigate relapse in the future

Relapse of the disease is thought to occur because of the failure of chemotherapy to eradicate LSCs. Targeting LSCs with novel agents will help to achieve a prolonged clinical remission. Development of new therapeutic strategies utilizing the survival (NF-kB, STAT, PI3 kinase, PTEN, p53) and self-renewal (Wnt, Hedgehog, Notch) pathways for the eradication of these dormant LSCs has critical therapeutic importance for the ultimate goal of cure for AML (Fig. 1).

## Natural Products for targeting LSCs

The pivotal obstacle for developing an efficient therapy targeting stem cell is to distinguish the apoptotic stimuli that can effectively target the LSCs while sparing normal hematopoietic stem cells in the milieu of an uncharacterized in vivo microenvironment. The highly proliferative bulk of AML population can be effectively targeted by conventional chemotherapy, while specific strategies designed to target this stem cell microenvironment may be effective. Along with genes involved in the control of stem cell self-renewal, leukemic stem cells are known to express high levels of genes involved in anti-apoptotic mechanisms. As standard chemotherapy approaches may not effectively target the LSC population, blocking molecular pathways involved in cell survival and chemoresistance that is unregulated in these populations can induce apoptosis in a selective manner. Various studies with leukemia progenitor cells show that drugs which can target multiple pathways deregulated in the LSCs, such as NF-κB, PI3 kinase and STAT can produce a significant apoptotic effect [72–75].

Natural products have been the mainstay of cancer chemotherapy for the past 50 years. Three quarters of currently available drugs are natural products or related to them [76]. More than 60% of the 140 anti-cancer agents approved since 1940 can be traced to a natural product. In 2000, 57% of all drugs in clinical trials for cancer were either natural products or their derivatives [77]. When compared with synthesized chemical compounds, natural products show a favorable profile in terms of their absorption and metabolism in the body with low toxicity. Natural products are also shown to possess multi-faceted mechanism that can hit multiple pathways that are de-regulated in cancer cells to achieve greater therapeutic efficacy.

### Parthenolide

**Fig. 2:**
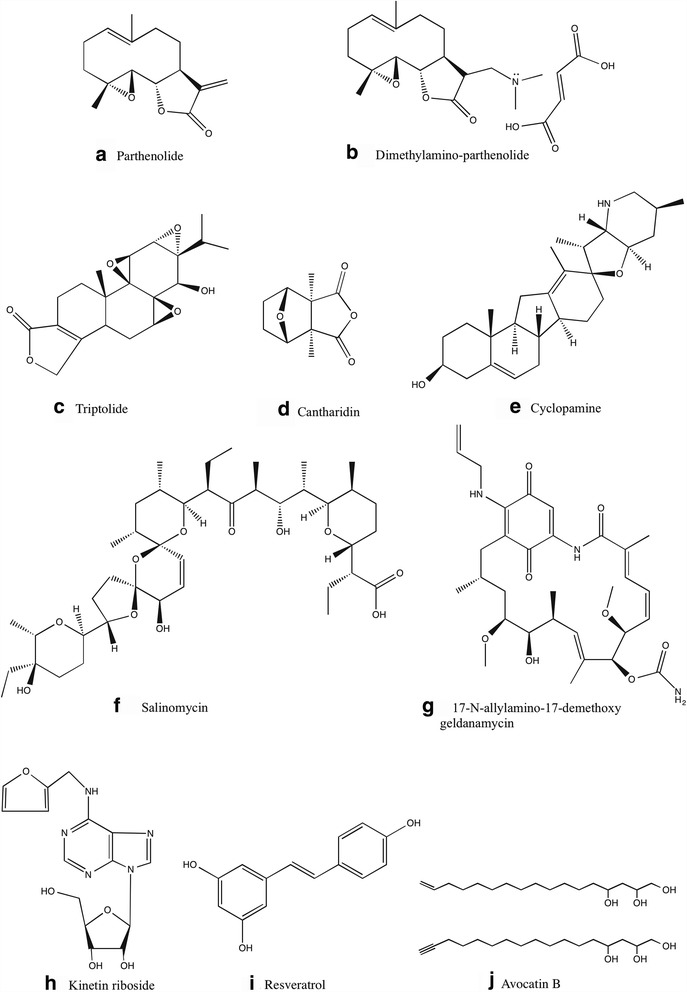
Chemical structure of various natural compounds that are shown to target AML stem cell population

Parthenolide (Fig. 2a), a naturally occurring sesquiterpene lactone containing an α- methylene-γ-lactone ring and an epoxide, belong to the germacranolide class and is present in the medicinal plant *Tanacetum parthenium* (feverfew) [78]. Parthenolide was reported to induce robust apoptosis in total as well as more primitive CD34^+^ populations from primary human AML specimens representing different French-American-British (FAB) subtypes while sparing normal hematopoietic cells. A side-by-side with the standard chemotherapy drug (cytarabine) showed that cytarabine was more toxic than parthenolide for normal cells with reduced toxicity to AML stem (CD34^+^/CD38^-^) and progenitor cells. Parthenolide was also shown to preferentially target AML progenitors (in vitro colony assay) and stem cell population in SCID mice xenograft model through the inhibition of NF-κB, proapoptotic activation of tumor suppressor p53, and increased reactive oxygen species (ROS) production [27].

Even though parthenolide is very effective in inducing AML LSC-specific cell death, its poor pharmacologic properties limit its clinical application. According to a Phase I dose escalation study of feverfew with standardized doses of parthenolide in patients with cancer, doses up to 4 mg as a daily oral capsule resulted in parthenolide plasma level well below detection limit of 0.5 ng/ml [79]. Recent studies on chemically modified parthenolide analogue, dimethylamino-parthenolide (Fig. 2b), have shown an oral bioavailability of ~70% compared with intravenous administration in mouse and canine models with an improvement in selectively eradicating AML stem and progenitor cells [80]. Dimethylamino-parthenolide also showed potent in vivo biological activity in spontaneous canine acute leukemia models and is currently evaluated in a Phase II clinical trial in AML patients. Further refinement in the bioavailability and selective toxicity will lead the way to a promising therapeutic drug.

Recent study by Pei et al. [81] shows that a parthenolide-based drug regimen containing parthenolide, 2-deoxyglucose and temsirolimus is a potent method of targeting AML stem cells while having no adverse effect on normal stem cells.

### Triptolide

Triptolide (Fig. 2c) is a bioactive diterpenoid triepoxide found in commonly used traditional Chinese medicinal plant, *Tripterygium wilfordii* (Thunder God Vine). Recent studies show that triptolide has a broad-spectrum anti-cancer activity against various hematological malignancies and solid tumors (IC_50_ of 2.6–50 nM in vitro and effective tumor inhibition in xenograft mice models at 0.15– 3 mg/kg in vivo), resulting in inhibition of tumor growth and inducing tumor cell apoptosis [82]. Due to toxicity and narrow therapeutic window, triptolide has been classified as an anticancer agent with limited therapeutic applications. Meanwhile, there are reports which suggest that triptolide might be useful as a promising chemotherapy sensitizer if it is used at low doses. It was used to enhance the cytotoxicity of conventional anticancer drugs (5-fluorouracil [83], cisplatin [84], dexamethasone [85], hydroxycamptothecin [86], etc) at low doses with limited adverse effects. Liu et al. [87] have reported that low-dose triptolide in combination with idarubicin induces apoptosis in LSC-like cells derived from KG1a cell line. KG1a cell line is derived from a male AML patient, in which most of the cells are still CD34^+^/CD38^-^, and they do not spontaneously differentiate into granulocytes and macrophage-like cells. The effects of triptolide on LSC-like cells is mediated through ROS generation, downregulation of Nrf2 pathway and HIF1α pathways.

### Cantharidin

Cantharidin (Fig. 2d), a natural toxin of terpenoid class secreted by many species of blister beetles, is used as a medicinal agent to remove warts. It is not a substrate for the multidrug resistant pumps in LSCs, hence it is a promising compound for selectively targeting LSCs. Cantharidin and its derivative Norcantharidin were found to inhibit hepatic leukemia factor, a gene implicated in the regulation of HSCs, and is also overexpressed in LSCs [88]. In vitro, Cantharidin and Norcantharidin were found to specifically target primary AML stem and progenitor cells by regulating the expression of genes involved in survival pathways such as SLUG, NFIL3 and c-myc, thereby inducing p53 and mitochondrial-caspase cascade to induce apoptosis. The dose-limiting toxicity of Cantharidin and Norcantharidin in vivo, limit their clinical application [89]. Nonetheless, potent in vitro LSC activity of cantharidin can be exploited clinically with the synthesis of new derivatives with reduced toxicity and in combination use with a suitable chemotherapeutic agent.

### Cyclopamine

Cyclopamine (11-deoxojervine) (Fig. 2e), naturally occurring steroidal jerveratrum alkaloid, is a teratogen isolated from the *Veratrum californicum* (corn lily). In AML, primary CD34^+^ blasts and CD34^+^ cell lines had a greater degree of hedgehog signaling pathway activation when compared to CD34^−^ blasts. Treatment with cyclopamine or monoclonal antibody to neutralize Hh ligands, result in Hh inhibition inducing apoptosis in CD34^+^ cell lines as well as sensitizing them against cytarabine. Cyclopamine treatments failed to affect growth or survival of AML cell lines without the G protein-coupled receptor, Smoothened (SMO), indicating the specificity of cyclopamine [56].

### Salinomycin

Salinomycin (Fig. 2f), a monocarboxylic polyether antibiotic, coccidiostat and ionophore with a preference for potassium, is isolated from *Streptomyces albus* [90]. Gupta et al. [91] reported that salinomycin can selectively kill human breast cancer stem cells. Salinomycin induces apoptosis and overcomes chemoresistance in LSCs and other tumor cells with ABC transporter-mediated multidrug resistance [92]. Human AML cell line KG1a treated with phenylbutyrate (histone deacetylase inhibitor) resembles characteristics of LSCs and display resistance to various chemotherapeutic drugs. Salinomycin treatment overcomes ABC transporter-mediated multidrug resistance to apoptosis-inducing concentrations of bortezomib and doxorubicin in human leukemia stem cell-like KG1a cells [93]. Salinomycin did not permit long-term adaptation and development of resistance of KG1a AML stem-like cells, which is an indictor of its value in clinical application. The proposed mechanisms of action include ROS generation, activation of Wnt/beta-Catenin pathway, inhibition of oxidative phosphorylation, cytoplasmic and mitochondrial K^+^ efflux, interference with ABC transporters and inducing differentiation of stem cells.

### 17-N-allylamino-17-demethoxy geldanamycin (17-AAG)

17-AAG (Fig. 2g) is a derivative of the antibiotic geldanamycin. It interacts reversibly with the ATP binding domain of HSP90 that is critical for its chaperone function, ultimately inducing tumor cell death [94]. 17-AAG was reported to preferentially induce apoptosis and eliminated the colony formation capacity of human AML LSCs. Treatment with low concentrations of 17-AAG selectively eliminates AML stem cells in vitro and in vivo by disrupting HSP90 client protein, HIF1α, while it failed to eradicate highly proliferative non-LSC terminal blast cells having constitutively active Akt-GSK3 signaling pathway [95].

### Kinetin riboside (6-Furfurylaminopurine riboside)

Kinetin riboside (Fig. 2h), a natural compound present in the coconut milk, is an anti-proliferative agent belonging to the ‘Cytokinins’ class of plant hormones. Kinetin riboside treatment results in CDKN1A upregulation, ATP depletion, cell cycle arrest at the G_2_/M phase, disruption of mitochondrial membrane potential, release of cytochrome c, caspase-3 activation, upregulation of Bad and down-regulation of Bcl-2 [96–98]. In vitro, Kinetin riboside induces apoptosis in the CD34^+^/CD38^-^ AML stem cell fraction and prevents LSC engraftment in NOD/SCID mouse model while sparing HSC fractions [99].

### Resveratrol

Resveratrol (Fig. 2i) (3,5,4′-trans-trihydroxystilbene) is a polyphenolic phytoalexin that has anti-oxidant, anti-inflammatory, cardioprotective, and anti-tumor activities [100]. It is structural similarities to estradiol and diethylstilbestrol and is present in the skin of red grapes, red wine, cranberries, blueberries and various other fruits. Resveratrol can inhibit growth and induce apoptosis in several human cancer cells, including mouse and human leukemia cell lines, through various mechanisms such as; modulating nitric oxide production, accumulation of p53 and p21, inhibition of ribonucleotide reductase and DNA polymerase, inducing arrest at the S and G_2_ phases of the cell cycle and inhibiting interleukin-1β–induced activation of NF-κB [101]. Hu et al. reported that resveratrol can selectively inhibit the growth of leukemia stem cell-like KG1a cells and sensitize cells to cytolysis by cytokine-induced killer cells through upregulation of NKG2D ligands (ULBP1, ULBP2 and ULBP3) and TNF-related apoptosis-inducing ligand receptors (DR4) [102].

### Avocatin B

Avocatin B (Fig. 2j), an Avocado (Persea americana) derived lipid, is a combination of two 17-carbon lipids (16-Heptadecene-1,2,4-triol & 16-heptadecyne-1,2,4-triol; 1:1 ratio) isolated from the methanolic extract of peel and seed of unripe avocado fruit. Cytotoxic property towards lung carcinoma, mammary adenocarcinoma, kidney carcinoma and pancreatic carcinoma cell lines has been reported by Oberlies et al. [103], with selectivity towards PC-3 human prostate adenocarcinoma cells, as potent as Adriamycin. It is also reported as an effective insecticide against yellow fever mosquito larva. Avocatin B was found to reduce viability of human primary AML progenitor and stem cells, while having no significant effect on normal peripheral blood CD34^+^ stem cells. Avocatin B induces ROS-dependent, mitochondria-mediated, apoptosis in AML cells, characterized by the release of apoptosis-inducing factor and cytochrome c in to the cytosol. It also inhibited fatty acid oxidation and decreased NAD and NADPH levels [104]. Avocatin B was also found to synergize other chemotherapeutics (cytarabine and doxorunicin) to induce leukemia cell death [105].

## Conclusion and perspectives

AML is a lethal form of hematologic malignancy, typically of stem or progenitor cell origin. The main hurdle to treat and cure AML is the inability to efficiently target and eliminate leukemia stem cells. Importantly, LSC stands apart from more differentiated blast cells with distinct set of unique biological properties and in most cases are not effectively targeted by standard chemotherapy agents, which can effectively kill leukemic blast cells in majority of patients. Due to the pivotal role of stem cells in the genesis, perpetuation and clinical relapse of AML, recent studies were focused on characterizing the molecular properties of LSC population that could be used for selective induction of apoptosis. Molecular analysis of AML LSC population shows that survival signaling mediated by NF-κB, STAT, PI3 kinase pathways and self-renewal regulatory pathways such as Wnt/beta-catenin, Hedgehog and Notch signaling pathways represent potential targets for therapeutic intervention. Similarly, re-activation of p53-mediated apoptosis pathways have shown to induce apoptosis in the LSC population. These findings demonstrate that more durable remissions in AML can be accomplished using a combination of selective inhibitors of above-mentioned survival pathways in LSC along with traditional regimens. Recent research advancement in the understanding of LSC bestows an expanding list of strategies to target LSC and some of the natural products summarized above (Table 1) have already been tried and proven to be effective. Moreover, combinations of natural products with chemotherapeutic drugs have been shown to preferentially induce apoptosis in human LSC, which is promising. The dandelion phenomenon predicts that treatments, which selectively attack stem cells, will not immediately eliminate the differentiated tumor cells and require a longer treatment period to obtain clinical responses compared to conventional therapies targeting the bulk population [106]. Therefore, LSC directed therapies utilizing natural compounds alone, may need longer treatment duration to be effective while use in combination with standard drugs like cytarabine and daunorubicin produce an early response to reduce the tumor cell population. A rational design of parthenolide-based drug regimen based on proteomic, genomic, and metabolomic methods by Pei et al. [81] is a perfect example of comprehensive approach to develop natural products based anticancer drug regimens. Similar studies based on the LSC validated natural products can offer numerous possibilities for eradication of AML stem cells, which can be translated to the clinical system. Also more natural products need to be screened for their ability to selectively target the LSCs. Natural products may also serve as models for the preparation of more efficacious analogues using chemical methodology such as total or combinatorial synthesis, or manipulation of biosynthetic pathways.

**Table 1:** Mechanism of action of various natural products on AML stem cell population

Compounds	Class of compound	Target and mechanism	Reference
Parthenolide	Sesquiterpene lactone	AML progenitors and CD34^+^/CD38^-^ stem cell population. In vivo and in vitro. Inhibition of NF-κB, activation of tumor suppressor p53, and increased production of reactive oxygen species (ROS).	[27]
Triptolide	Diterpenoid triepoxide	CD34^+^/CD38^-^ stem-like cells derived from KG1a cell line. In vivo and in vitro. Generation of ROS, downregulation of Nrf2 and HIF-1α pathways.	[87]
Cantharidin	Terpene	Primary AML stem (CD34^+^) and progenitor cells. In vivo and in vitro. Modulate expression of genes involved in survival pathways (HLF, SLUG, NFIL3 and c-myc), induces p53 and mitochondrial-caspase cascade.	[89]
Cyclopamine	Steroidal jerveratrum alkaloid	CD34^+^ cell lines and primary CD34^+^ AML stem cells. Inhibition of aberrantly activated hedgehog pathway and Bcl-2 expression.	[56]
Salinomycin	Monocarboxylic polyether antibiotic	CD34^+^ CD38^−^ KG1a AML SCs expressing functional ABC transporters P-glycoprotein, ABCG2, and ABCC11. In vitro. Overcomes ABC transporter-mediated multidrug and apoptosis resistance.	[93]
17-N-allylamino-17-demethoxy geldanamycin (17-AAG)	Geldanamycin derivative	Human AML CSCs (CD34^+^ CD38^−^) and murine lymphoma CSCs (c-Kit^+^Sca1^+^). In vitro. Induce apoptosis and eliminate the colony formation capacity by disrupting the transcriptional function of HIF1α.	[95]
Kinetin riboside	Cytokinin	CD34^+^CD38^−^ cell fraction present in primary AML samples. In vivo and in vitro. Induces apoptosis in LSCs and prevents AML stem cell engraftment in NOD/SCID mouse model. Adenosine kinase activity is required for the anti LSC effect.	[99]
Resveratrol	Polyphenolic phytoalexin	AML stem-like KG1a cells. In vitro. Upregulate expressions of NKG2D ligands (ULBP1, ULBP2 and ULBP3) and TRAIL receptor 1 leading to enhanced cytokine-induced killer cell-mediated cytolysis.	[102]
Avocatin B	17-Carbon lipid	Primary AML stem (CD34^+^) and CD34^+^ cell lines. In vivo and in vitro. Generation of ROS, mitochondria-mediated apoptosis, characterized by the release of apoptosis inducing factor and cytochrome c.	[104]

## Abbreviations

AML: Acute myeloid leukemia
CD: Cluster of differentiation
HSC: Hematopoietic stem cells
LSC: Leukemia stem cells
NF-κB: Nuclear factor kappa-light-chain-enhancer of activated B cells
PI3K: Phosphatidylinositol-4,5-bisphosphate 3-kinase
ROS: Reactive oxygen species
STAT: Signal Transducers and Activators of Transcription
